# Formulation of Tolmetin Ocuserts As Carriers for Ocular Drug Delivery System

**Published:** 2017

**Authors:** Parastoo Tofighia, Saeeide Soltani, Seyed Hassan Montazam, Seyed Ataollah Montazam, Mitra Jelvehgari

**Affiliations:** a *Faculty of pharmacy, International Aras Branch, Tabriz University of Medical Sciences, Tabriz, Iran.*; b *Drug Applied Research Center, Tabriz University of Medical Sciences, Tabriz. *; c *Department of Microbiology, Bonab Branch, Islamic Azad University, Bonab, Iran.*; d *Student Research Committee, Ardabil University of Medical Sciences, Ardabil, Iran.*

## Introduction

Ocular drug delivery is one of the most intriguing and challenging endeavors pharmaceutical scientists have faced during the past 10-20 years (1). For the ophthalmic drug-delivery systems, the physiological restrictions imposed by the protective mechanisms of the eye results in the low absorption of drugs leading to the short duration of action (2). The bioavailability of conventional ocular drug delivery systems like eye drops is little observed, since eye is protected by a number of complex defense mechanisms that restricts achieving an effective drug concentration within the target area of the eye (2).

For ophthalmic use, a number of new preparations have been developed in order not to prolong the contact time of the vehicle on the ocular surface, but to reduce the drug elimination speed. Successful results have been recorded for inserts. Another approach to optimize the bioavailability is the implementation of mucoadhesive concept. To this end, suitable polymers come into interaction with the mucus layer and coat the external surface of the eye (3).

Advantages of ocular drug delivery systems are: easy, convenient, and needle free drug application without need for trained personnel assistance for the application, and self medication; thus improving patient compliances compared to parenteral routes. Good penetration of hydrophilic, low molecular weight drugs can be obtained through the eye. Further, rapid absorption and fast onset of action is achieved because of large absorption surface area and high vascularisation. Ocular administration of convenient drugs would therefore be influencing in emergency therapy as an alternative to other administration routes. Accordingly, avoidance of hepatic first pass metabolism and thus potential for dose reduction is influencing compared to the oral delivery (4). 

A series of approaches have been developed to tackle the problem in recent decades, of which colloidal drug delivery system has attracted much attention. As an example, sol to gel phase transition on ocular surface such as carbopol and hydroxypropylmethyl cellulose have been recognized as effective ocular drug carries (5). This problem can be solved by applying gel systems and ophthalmic inserts. Gel is knows as a network of polymer chains that are water-insoluble, sometimes found as a colloidal gel in which water is the dispersion medium. Gels are superabsorbent (containing more than 99% water) natural or synthetic polymers. They also possess a degree of flexibility very akin to natural tissue, owing to their significant water content (5). Gel systems can be formulated in a liquid phase suitable to be administrated by instillation into the eye cavity. Upon exposure to the stimuli such as pH, temperature, ion activation, etc. they change to the gel phase and thus improve the residence time and corneal bioavailability of the drug.

Ocular inserts films (as erodible gels) are the most logical delivery systems that are aimed to remain for a long period of time in the front of eye. These polymeric delivery systems do the sustaining and controlling drug release and thus avoiding pulsed entry characterized by a transient overdose. Then process is followed by a relative short period of acceptable dosing, which is in turn followed by a prolonged period of under dosing (5). Inserts have been a success in the improvement of accurate dosing, and drug bioavailability, the reduction of systemic absorption, and consequently side effects.

Topical administration of drugs is the most preferred route that manages the ocular inflammations as it provides higher ocular drug concentrations and avoids the systemic side effects associated with the oral administration. However considering the physiologic constraints of the eye, only few anti-inflammatory agents representing particular physicochemical properties could be formulated in an appropriate dosage form that could be effective for the management of ocular inflammations (6). In increasing evidence, prostaglandins have been recognized as leading mediators of the inflammatory process in the eye and in other tissues, as well. They have been clearly implicated in postsurgical inflammation and anterior uveitis and are possibly responsible for most of the clinical features seen in these conditions (6). The recognition of important role of prostaglandins has led to an interest in the use of non-steroidal anti-inflammatory agents as potential alternatives to the long established steroid therapy.

One such non-steroidal anti-inflammatory drug that has had widespread usage as a systemic treatment for the rheumatoid arthritis and allied conditions is tolmetin (Tol). This compound, which is a pyrrole acetic acid derivative without indole nucleus present in indomethacin, is a potent inhibitor of prostaglandin synthesis and has been developed as an ophthalmic solution. Tolmetin is highly effective as a topical anti-inflammatory agent in an experimental model of immunogenic uveitis. Tolmetin ophthalmic solution has also been shown to be fruitful in comparison with saline, in a small group of patients treated after cataract surgery (6).

The ocular anti-inflammatory activity of aqueous Tol (0.5%) ophthalmic solution was assessed and compared with a mucoadhesive formulation of Tol in sodium arachidonate-induced ocular inflammation in rabbits. The treatment with Tol resulted in the significant reduction of the signs and symptoms of ocular inflammation. A significant reduction in sodium arachidonate-induced aqueous humor PGE2 levels, polymorphonuclear leukocytes, protein concentration and IOP rise was also observed in eyes treated with Tol . The results of the pharmacokinetic evaluation represented significantly higher aqueous humor drug concentrations in eyes inflamed as compared to uninflamed eyes. Furthermore, the mucoadhesive formulation of Tol provided 2.6- and 2.0-fold higher aqueous humor area under the concentration-time curve (AUC) values compared to the aqueous formulation in uninflamed and inflamed eyes, respectively (6) .

Typical polymers that have been used as mucoadhesive drug carriers are poly acrylic acid, poly methacrylic acid, cellulose derivatives, poly ethylene oxide, lectin and chitosan, PAA and its cross-linked commercial forms.

Hydroxypropyl methylcellulose (HPMC) is an odorless and tasteless, white to slightly off-white, fibrous or granular, and free-flowing powder that is a synthetic modification of the natural polymer, cellulose (7). This polymer rarely interacts with colorants. HPMC closely approaches the desired attributes of an ideal. When used alone, the polymer has the tendency to bridge or fill the debossed tablet surfaces. A mixture of HPMC with other polymers or plasticizers is utilized to resolve the bridging or filling problems (7).

Several polymeric systems are worked out to fabricate ocular inserts for better ocular bioavailability and retention of drugs. Ocular *in situ* gelling systems offer the advantage of convenient administration and increased contact time. These systems undergo sol-to-gel phase transition at ocular surface due to main three mechanisms, namely pH triggered system such as carbopol (3).

Cross-linked polyacrylic acid shows excellent mucoadhesive properties that cause significant enhancement in the ocular bioavailability. Carbopol 934 P is a high cross link water swellable acrylic polymer and has an approximate molecular weight of 3000000 Da. which make it appropriate to be used in pharmaceutical industry. Park Robinson and Ponchel *et al.* reported in their study that poly acrylic acid interacted with functional group of mucus glycol protein via carboxylic group (8). 

Carbopols, which are acrylic acid polymers with very high molecular weight, have been used mainly in liquid or semi-solid pharmaceutical formulations, such as gels, suspensions and emulsions, as a thickening agent, in order to modify the flow characteristics. Recently, they have also been used for their mucoadhesive properties. Carbopol 934P (CP) is a mucoadhesive polymer which has been investigated as a useful adjuvant in bioadhesive drug delivery system. The principal reasons for the addition of mucoadhesive polymers to the system are the possibilities of prolongation of residence time in organ and in turn increase of the contact time with absorbing mucosa, thereby resulting in the enhancement of drug absorption. CP is a mucoadhesive polymer and has been investigated widely by the pharmaceutical industry due to its high viscosity at low concentration and low toxicity (8). CP is a polyacrylic acid polymer, which is cross linked with allyl sucrose. This acidic carboxylic group partially dissociates in aqueous solution, producing a flexible coil structure. It is a fact that the carbopol gels are prepared by dispersing polymer in water, in which it swells up to 1000 times of the original volume while neutralizing the system. It allows the ionization of carboxylic groups, and in result, a strong gel is formed. The gel formation of CP is dependently associated the electrostatic repulsion between the anionic carboxyl groups. In aqueous solution, the CP gel shows the following behaviors: 1) the gel consists of closely packed swollen particles; 2) the gel forms a thick layer that inhibits water penetration (8).

**Table1:** Tolmetin films prepared by solvent casting method with different drug and polymers (HPMC and HPMC with carbomer 934p) ratio

**Formulation code**	**Drugs to polymer ratio**	**Tolmetin** **(mg)**	**HPMC** **(mg)**	**carbomer 934p (mg)**	**Ethanol** **(mL)**	**Propylen glycol (g)**	**glycerol** **(g)**	
T1	1:3	200	600	-	20	0.05	-
T2	1:4	200	800	-	20	0.05	-
T3	1:5	200	1000	-	20	0.05	-
T4	1:6:2.5	100	600	250	20	-	0.03
T5	1:8:2.5	100	800	250	20	-	0.03
T6	1:10:2.5	100	1000	250	20	-	0.03

a T_1_ to T_3_ formulations prepared with HPMC polymer alone and T_4_ to T_6_ formulation prepared with HPMC and Carbomer 934p polymers.

b Plasticizers of propylene glycol applied for T_1_ to T_3 _formulations and glycerol for T_4_ to T_6_ formulations.

**Table 2:** Effect of drug to polymer ratio on physicochemical characteristics and mucoadhesivity tolmetin films

**Variables**	**Formulation code**					
	**T**_1_	**T**_2_	**T**_3_	**T**_4_	**T**_5_	**T**_6_
**Drugs : Polymer ratio**	1:3	1:4	1:5	1:6:2.5	1:8:2.5	1:10:2.5
**Weight variation** (mg ± SD)	4.1 ± 0.23	6.5 ± 0.52	10.2 ± 0.95	9.10 ± 0.86	11.05 ± 0.80	13.95 ± 1.20
**Thickness** (µm ± SD)	62.6 ± 1.0	73.0 ± 1.0	83.2 ± 0.9	110.30 ± 0.03	123 ± 0.007	243 ± 0.05
**Folding endurance** (n ± SD)	> 300	> 300	> 300	> 300	> 300	89 ± 12
**Drug content** (mg/cm^2 ^± SD)	0.939 ± 0.22	1.115 ± 0.11	1.811 ± 0.4	1.03 ± 0.33	1.15 ± 0.60	1.23 ± 0.02
**Content uniformity** (% ± SD)	74.91 ± 2.36	85 ± 2.11	90 ± 5.91	100 ± 0.02	98.59 ± 0.02	96.63 ± 0.02
**Production Yield** (% ± SD)	85.5 ± 29.30	98.02 ± 11.80	100 ± 8.30	99.10 ± 9.1	100 ± 6.60	99.40 ± 2.30
**Absorbed moisture** (% ± SD)	7.32 ± 0.65	5.44 ± 0.21	4.96 ± 0.38	0.79 ± 0.44	1.06 ± 0.95	4.52 ± 1.79
**Loss moisture** (% ± SD)	1.89 ± 0.27	5.38 ± 0.01	6.20 ± 0.07	0.79 ± 0.04	0.73 ± 0.08	2.85 ± 0.66
**pH surface** (n ± SD)	5.92 ± 0.30	6.2 ± 0.40	6.0 ± 0.40	6.6 ± 0.2	6.8 ± 0.1	6.9 ± 0.1
**Swelling index** (% ± SD)	25.74 ± 0.05	21.88 ± 0.05	18.38 ± 0.05	19.85 ± 0.02	17.23 ± 0.01	14.18 ± 0.02
**Mucoadhesive** **strength** (g/cm^2 ^± SD)	139 ± 57	135 ± 21.8	128 ± 28	135 ±13	148 ± 84	161 ± 66
**Residence time** (Sec ± SD)	15 ± 0.02	15 ± 0.01	15 ± 0.00	25 ± 0.14	20 ± 0.81	30 ± 0.26

**Table 3.** Flux or amount of drug release per unit surface area after 4 h, Permeability coefficient, intercept and regression coefficient for different formulation and comparison of various release characteristics of tolmetin from different film formulations

**Formulation** ** code**	**Flux** **(mg/cm** ^2^ .min)*10 ^-3^	**Permeability coefficient** (cm/min) *10 ^-3^	**Intercept** **mg/cm** ^2^ **)** **)**	**r** ^2^
T_1_	1.6	40.89	-0.0207	0.9817
T_2_	1.3	27.98	-0.0297	0.9938
T_3_	0.9	11.93	-0.0135	0.9891
T_4_	0.6	13.98	0.0531	0.9924
T_5_	0.4	8.35	0.0469	0.9849
T_6_	0.3	5.85	0.0589	0.9994

The objective of the present work was to provide controlled and continuous drug delivery. Ocular therapy in the anti-inflammatory effect would be significantly improved if the precorneal residence time of drugs could be increased. Furthermore, a relevant amount of work has been performed on the bioadhesive potential of carbopol and HPMC polymers.

## Experimental

*Materials and methods*

*Materials*

Tolmetin sodium (Medichem, China), hydroxypropylmethyl cellulose (Sigma-Aldrich, USA), Carbopol 934P (B.F.G, USA), ethanol, propylene glycol, glycerol, ethanol, sodium hydroxide, buffer phosphate (pH 6.8), sodium chloride, potassium chloride, sodium sulfate, ammonium acetate, urea, lactic acid, and liquid paraffin were purchased from Merck (Darmstadt, Germany). All solvents and reagents were of analytical grade.

*Method of film preparation*

 Tol films were prepared using HPMC /and cabomer 934p mixture with different concentration ratios of HPMC alone and with cabomer 934p by solvent casting technique (Table 1). Accurately weighed quantity of HPMC was soaked in 20 mL ethanol for 24 h, calculated amount of 200 mg Tol was dissolved in the polymeric solution, and propylene glycol was added gradually with continuous stirring. Then 20 mL resultant mixture was poured into each fabricated glass ring placed in a petri dish, and then it was put aside for drying at room temperature for 24 h. The dried polymeric films were cut into circular films of 10 cm diameter for further evaluation. The same films were prepared by using HPMC and cabomer 934p in different concentration ratios and glycerol was added as plasticizer (Table 1).These new films were examined in order to identify and select the film having the best characteristics.

*Characterization of film studies*

Appearance of the films was appraised by observing the color, elegance, stickiness and texture.

*Weight of films*

For evaluation of film weight, six films of every formulation (1×1 cm^2^) were taken and weighed

individually on a digital balance (Sartorius GmbH, , Germany) and average weights were calculated.

*Folding endurance*

Three films were cut from each formulation with the size of 1×1 cm^2^. Folding endurance of films were determined by repeatedly folding of the film at the same place till it broke or folded up to 300 times without breaking which gave the value of folding endurance of the film 

(9). 

*Thickness of the films*

The thickness of prepared films was determined by using digital vernier calipers at five different points (at center and four corners) of the film and the average was calculated (Mitutoyo, Japan) (10).

*Determination of surface pH*

Inserts were left to swell for 5 h on agar plate prepared by dissolving 2% (w/v) agar in warm simulated tear fluid (STF; sodium chloride: 0.670 g, sodium bicarbonate: 0.200 g, calcium chloride. 2H _2_ O: 0.008 g, and purified water q. s. 100 g) of pH 7.4 under stirring and then pouring the solution into petri dish till gelling at room temperature. After the time of soaking, the pH of wet surface was measured by placing the electrode in contact with the surface of 

insert (11).

*In-vitro swelling studies*

Swelling of the polymer depends on the concentration of polymer, ionic strength, and the presence of water. Initial diameter of films (1x1 cm^2^) were measured individually (D_1_) and placed separately in petri dishes containing 5 mL of phosphate buffer (pH 6.8) solution. 

The dishes were stored at room temperature. Then at regular intervals (up to 1 h), swollen film diameter was re-measured (D_2_) and the swelling index was calculated by the following formula (12):

Swelling index = D_2_-D_1_/D_1_

*Moisture content loss and moisture absorption*

The films were accurately weighed and kept in desiccators containing: a) anhydrous calcium chloride and b) 100 mL of saturated solution of aluminum chloride, which maintains 76% and 86% humidity (RH). After three days, the films were taken out and weighed. The moisture content (%) was determined by calculating moisture loss (%) using the formula (13):

Moisture content (%) =initial weight – final weight / initial weight × 100

The moisture absorption was also calculated using the following formula (13):

Moisture absorption (%) = final weight - initial weight / initial weight ×100 

*Drug content and content uniformity*

The films (six samples of each film) were analyzed for the content uniformity by dissolving 1×1cm^2^ films in 10 mL STF with pH 7.4 to simultaneous shaking for several hours. The absorbance of the solution Tol was measured by UV spectrophotometer at 318.6 nm. All experiments were performed in triplicate.

*Differential Scanning Colorimetry (DSC)*

Differential scanning calorimetry (DSC) monitors heat effects associated with phase

transitions and chemical reactions as a function of temperature. In a DSC the difference in heat flow to the sample and a reference at the same temperature, is recorded as a function of temperature. The reference is an inert material such as alumina, or just an empty aluminum pan. The temperature of both the sample and reference are increased at a constant rate. The physical state of drug in the films was analyzed by Differential Scanning Calorimeter (Shimadzu, Japan). The thermograms were obtained at a scanning rate of 10 °C/min conducted over a temperature range of 25-300 °C.

*Bioadhesion strength*

The tensile strength demanded to detach the bioadhesive films from the mucosal surface was applied as a measure of the bioadhesive performance. The apparatus was locally assembled. The device was principally composed of a two-armed balance (Figure 1). The mucoadhesive forces of films were determined by means of the mucoadhesive force-measuring device (14), using the sheep cornea eye. Whole eye bulbus of an adult sheep was obtained from a local slaughter house, and the underlying skin was removed to obtain freshly excised conjunctiva. The pieces of ocular mucosa were placed in isotonic sodium phosphate buffer with pH 7.4, at 37 ˚C ± 1˚C. At the time of testing, a section of mucosa was secured to the upper glass vial (C) using a cyanoacrylate adhesive (E). The diameter of each exposed mucosal membrane was 1.5 cm. One vial with a section of tissue (E) was connected to the balance (A) and the other vial was fixed on a height-adjustable pan (F). To expose the tissue on this vial, a constant amount of films (D) was applied. The height of the vial was so adjusted that the films could adhere to the mucosal tissues of both vials. Immediately, a constant force of 0.5 N was applied for 2 min to ensure the intimate contact between the tissues and the samples. The vial was then moved upwards at a constant speed and connected to the balance. Weights were added at a regular rate to the pan on the other side of the modified balance of the used device until the two vials were separated. During measurement, 150 μL of sodium phosphate buffer with pH 7.4 was evenly spread onto the surface of test membrane. The bioadhesive force, as the detachment stress mentioned in g/cm^2^, was determined from the minimal weights that detached the tissues from the surface of each formulation using the following equation (14): 

$\mathrm{Detachment stress}(g/\mathrm{cm}2)=\frac{m}{A}$

Where *m* is the weight added to the balance in grams and *A* is the area of tissue exposed. Measurements were repeated three times for each of the films. All the above three experiments were conducted in triplicate. 

*Ex-vivo mucoadhesion time*

The selected batch was subjected to *ex vivo* mucoadhesion test. The disintegration medium was composed of 900 mL phosphate buffer pH 6.8 maintained at 37 ^°^C. The sheep cornea eye, 3 cm long, was glued to the surface of a glass slab, vertically attached to the disintegration apparatus (Erweka, Germany) (15). The mucoadhesive discs were hydrated from one surface and then were brought into contact with the mucosal membrane. The glass slab was vertically fixed to the apparatus and allowed to move up and down so that the disc was completely immersed in the buffer solution at the lowest point and was out of solution at the highest point. The time necessary for complete erosion or detachment of the discs from the mucosal surface was recorded. The experiment was carried out in triplicate.

*Ex-vivo transcorneal permeation studies*

The *in-vitro* permeation study of the Tol films through the cornea of eye was performed using Franz diffusion cell at 34 ± 0.2 °C. Freshly obtained scleral layer was mounted between the donor and the receptor compartments. The films were placed on the epithelial faced surface and the compartments were clamped together. The film sized 1×1 cm^2^ was placed on the cornea and opening of the donor compartment was sealed with a glass cover slip and soaked with 7-10 µL simulated tear fluid (STF, composition: NaCl 0.68 g, NaHCO3 0.22 g, CaCl2.2H2O 0.008 g, KCl 0.14 g, and distilled deionized water to 100 mL). The receptor compartment was filled with 22-25 mL STF, pH 7.2, and stirred with a magnetic bead at 20 rpm to simulate blinking action (14). Three milliliters of sample were withdrawn at predetermined time intervals and analyzed for drugs at 323 nm.

Permeability coefficient was calculated using the following equation:

$K_{p}=\frac{\mathrm{Jss}}{C_{{}^{\circ}}}$

where Jss is the steady state flux per unit area, Kp is the permeability coefficient for a given solute in a given vehicle (cm h^-1^), and is the concentration of the solute in the donor compartment. 

*Statistical analysis*

Where appropriate, release results were evaluated using a one-way ANOVA at 0.05 levels . 

## Results

*In-vitro* characterization studies

In the present research, Tol mucoadhesive films were prepared using different combinations of different concentration ratios of HPMC alone and HPMC with carbomer 934p by solvent casting technique to improve the efficacy of drug by improving its bioavailability. 

*Weight of films*

The prepared films were evaluated for various parameters. The results are given and discussed in the following sections (Table 2).

*Thickness of the films*

The thickness of films prepared with various polymer combination ratios was found to be in the range of 113-145 µM, suggesting that the films were thin enough and they would not cause any inconvenience after their application to the ocular cavity (Figure 2). 

*Folding endurance*

Tol films prepared with HPMC polymer with CP did not show any cracks even after folding for more than 200-300 times; hence it was taken as the end point. The values were observed to be optimum to reveal good film properties. 

*Surface pH*

The surface pH of all the films was found to be in the range of 5.42-6.77, a range that is nearer to the ocular pH (6-7.6), hence it can be safely assumed that the films when applied to any mucus membrane of the ocular cavity would not cause any irritation. pH of all formulations were safe and non-irritating to oral mucosa (Table 2). 

*Swelling studies*

All films showed high diameter swelling and the recorded swellings after 2 h were 90-180% (for HPMC alone films) and 50-100% (for HPMC combined with CP films).

*Moisture content loss and moisture absorption*:

The percentage of moisture absorption was shown to range between 1.25±0.05 and 7.25±1.38 for HPMC alone films and 17.79±1.44 and 34.55±2.79% for HPMC combined with CP films. The moisture loss, 7.22-13.12% (for HPMC alone) and 5.45-10.32% (for HPMC with CP films), is shown in Table 2. Hence, the high moisture absorbing capacity was detected in T_3_ (7.25%) and T_6_ (34.55%), and more moisture loss was observed in T_1_ (13.12%) and T_6_ (10.32%). 

*Drug content and content uniformity*

The content and content uniformity of Tol and HPMC alone films (T_1_-T_3_) were in the range of 2.64-2.90% and 98.13-107.08%, and of HPMC combined with CP films (T_4_-T_6_) were in the range of 1.96-2.32% and 74.91-87.8%, respectively, though there was a minor change in the loss of drug among the formulations (Table 2). The films were evaluated for drug content uniformity, which indicated that the drug was uniformly dispersed in the range of 74.44% and 107.07% for T_1_ and T_4_, respectively (9-12).

*DSC studies*

According of Figure3, it is possible that the drug has dispersed in crystalline or amorphous form or dissolved in the polymeric matrix during formation of the films. Any abrupt or drastic change in the thermal behavior of either the drug or polymer may be representative of a possible drug-polymer interaction (16). The endotherm peak of pure drug was observed at 160 °C (Figure 3). However in the thermogram of the films there was no endothermic peak of the drug melting up to 200 °C, suggesting the amorphous state of the drug in the films. CP showed a wide endothermic peak in the range of 44.42-107.21°C. HPMC also demonstrated a very wide endothermic peak at 41.59-86.24 °C which may be related to initial water content of the powder. Such a mass loss results in the baseline change as well. HPMC was melted up to 300°C and the exothermic peak (decomposition peak) was observed to be near 250 °C. 

*Ex-vivo mucoadhesive characterization studies*

*Mucoadhesion time*

The* in-vitro* residence time determined the period of adhering the films with HPMC alone (15-17 min) and HPMC combined with CP films to the mucosa and ranged from 21 to 48 min. The films when applied could be retained for a longer period of time at the site of application and this conclusion is well supported by the long *in-vitro* residence time of 48 min obtained for T_6_.

*Bioadhesion strength*

Folding endurance of the prepared film was measured to be 40-300 times and the mucoadhesive strength was found to be 1.28 g/cm^2^ and 1.61 g/cm^2^. The values for these parameters are high enough to indicate that the films were flexible and would not detach easily (10-12). 

*In-vitro permeation studies*

Figure 4 represents the comparison of permeation of HPMC alone films and HPMC combined with CP films through ocular mucosa for formulations containing a different drug-to-polymer ratio. Slopes of the linear portion of the release profiles were calculated. These slopes depicted the rate of release or flux of HPMC alone and HPMC combined with CP films from different formulations (Table 3). The highest fluxes and regression coefficient for T_1_ and T_4_ formulations were 0.0016 mg/cm^2^.min, 0.9817 (HPMC alone) and 0.0006 mg/cm^2^.min, 0.9924 (HPMC combined with CP), respectively.

## Discussion

Ophthalmic inserts offer many advantages over conventional dosage forms like: increased ocular residence, possibility of releasing drug at a slow and constant rate, accurate dosing and increased shelf-life. The physiochemical evaluation data presented in Table 2 indicates that the thickness of the ocular film varies. Films with HPMC alone showed less thickness of polymer film which is the desired quality of ocular films. These results indicated that the film forming polymer HPMC with propylene glycol on drying formed a thin and more transparent film as compared to HPMC with CP by using glycerin as plasticizer (Table 2). All the formulations (six sample of each film) exhibited uniform thicknesses with low standard deviation values ensuring the uniformity of the films prepared by solvent casting method (Table 2).

All Films (except T_6_) showed good folding endurance because polymer forms a hydrogen bond with the polymer molecule; thereby imparting flexibility to the film. The film containing HPMC and CP showed maximum mucoadhesion, while film containing HPMC alone showed less mucoadhesion (Table 2). The results showed that high viscosity of polymer exhibited higher adhesion and better mucoadhesive property in comparison to HPMC alone polymer at the same concentration. This may be due to the presence of numerous disulphide bridges and carboxyl and hydroxyl groups, which adopt more favorable macromolecular conformations, and accessibility of its hydrogen-binding groups when compared with other polymers. HPMC as a cellulose derivative, formed weaker bonds with mucus, which may be relevant to either a decrease in present hydrogen binding sites or unfavorable entanglement with the mucus; though CP made stronger bonds with the three-dimensional mucin network (17).

Due to the uniform dispersion of drug in the matrix of the polymer, a significantly appropriate volume of drug was loaded in all the formulations. The loss of drug could be in association with its aqueous insolubility. Tol is water-soluble and does not initiate settling down from medicated solutions when dispersed for removal of air bubbles. Therefore the solutions were casted as films possessing relatively thorough amount of drugs (18). 

The pH of films was in the range of 5.9 to 6.9. As the pH of ocular mucosa varies between 6.5 and 7.6 (19), HPMC with CP polymers was found to be suitable for preparing ocular films.

Table 2 shows the swelling profiles of prepared ocular inserts. HPMC films showed higher swelling or water uptake than HPMC with CP films during study, however the films reached equilibrium sooner. Increasing the CP in HPMC films decreased the swelling of films; this emphasizes the water retaining capacity of HPMC (20). On the other hand, HPMC with CP film showed lower swelling than HPMC film. Formulations T_1_ to T_3_ showed statistically non-significant differences (p > 0.05) in swelling with respect to formulations T_4_ to T_6_. The rate and the extent of insert hydration and swelling also influence the insert adhesion and finally the drug release from the insert (12, 14). Hence this parameter is important for predicting the drug release as well as the bioadhesive potential of matrix. Excessive hydration of a polymeric film could converge in a decrease in the adhesive strength and possibly the loss of adhesion and hence a shorter duration of retention (11). The solution of insert was kept at room temperature for 24 h to enhance the inter-diffusion of polymer particles. Upon drying, polymer solutions were converted into drug polymer inserts/films. 

Some researchers have studied the mechanism of film formation using polymer dispersions. The film formation accomplishes in three stages: (a) evaporation of the casting solvent and subsequent concentration of polymer particles, (b) deformation and coalescence of polymer particles, and (c) further fusion by inter-diffusion of polymeric molecules of adjacent polymer particles (21). The physical state of drug in the dried polymer is dependent on the solubility of drug in the polymer. For the present case, the drug was dispersed in the polymeric solution. The accomplishment of film formation method is further proved by the fact that the prepared inserts/films were translucent, colorless, and smooth in texture, uniform in appearance, thick, and weighty, and showing no visible crack or imperfection (11).

The percentage of moisture absorption is linked to the capacity of excipients to absorb water in vapor form. The utilized HPMC polymer is a hydrophilic polymer. It is hypothesized that the initial moisture content plays the role of a determining factor in the moisture absorption (22).Other films show primarily low moisture content as is shown by percentage of moisture lost. There is an inverse association between these two parameters; the higher is the percentage of moisture lost, the lower is the moisture absorbed and vice versa (23).

The DSC analysis of films clarified a significant change in the melting point of drug, revealing the modification or interaction between the drug and the polymer (Figure 3).

Tol drug, containing water-soluble molecules and allowing more water influx, leads to a quicker dissolution and erosion from the mucosal surface. HPMC is a hydrophilic polymer and may show more affinity towards mucin which comprises 95% water. This may be the reason for longer residence time (integrity of the films is shorter). Moreover, as reported in literature, the high erosion rate was observed with the non ionic polymers such as HPMC (24). 

Permeation data of Tol from ophthalmic film are represented in Table 3. The data reveals that the drug permeability decreases with the concentration of HPMC from 600 to 1000 mg. It is worth mentioning that an increase in the permeation was observed with decreasing the concentration of HPMC, though. Nonetheless, Sieg and Robinson (25) and Madhu *et al.* (26) also reported that corneal epithelium acted as a reservoir for drug accumulation and provided continuous delivery of the drug to the aqueous humor in context to permeation studies of Tol. The present study demonstrated the influence of different concentrations of HPMC polymers alone on their permeation rate.

## Conclusion

Ocular mucoadhesive film formulations containing Tol were prepared with satisfactory physicochemical characterizations. The release patterns and bioadhesion properties could be controlled by changing the polymer type and concentration. The present study indicated a good potential of the prepared mucoadhesive films containing Tol for ocular delivery with added advantages of circumventing the hepatic first pass metabolism, substantial dose and side effect reduction. This study confirmed the potential of the above film dosage forms as promising candidates for ocular delivery of the Tol.

## References

[1] Thakur RR, Kashiv M (2011). Modern delivery systems for ocular drug formulations: A comparative overview wrt conventional dosage form. Int. J. Res. Pharm. Biomed. Sci..

[2] Singh V, Bushetti S, Raju SA, Ahmad R, Singh M, Ajmal M (2011). Polymeric ocular hydrogels and ophthalmic inserts for controlled release of timolol maleate. J. Pharm. Bioallied Sci..

[3] Mishra D, Gilhotra R (2008). Design and characterization of bioadhesive in-situ gelling ocular inserts of gatifloxacin sesquihydrate. Daru J. Pharm. Sci..

[4] Gaudana R, Jwala J, Boddu SH, Mitra AK (2009). Recent perspectives in ocular drug delivery. Pharm . Res..

[5] Rathore K, Nema R (2009). An insight into ophthalmic drug delivery system. Int. J. Pharm. Sci. Drug Res..

[6] Ahuja M, Dhake AS, Sharma SK, Majumdar DK (2008). Topical ocular delivery of nsaids. AAPS J..

[7] Akbari J, Saeedi M, Morteza-Semnani K, Fallah-Vahdati G, Lesan N (2014). The effect of plantago major mucilage on the release profile and bioadhesive properties of propranolol hcl bucccoadhesive tablets in comparison with current polymers. Pharm. Sci..

[8] Jelvehgari M, Rashidi MR, Samadi H (2006). Mucoadhesive and drug release properties of benzocaine gel. Iran . J. pharm. sci..

[9] Drug BMFCA (2011). Journal of chemical and pharmaceutical research. J. Chem..

[10] Rajput G, Majmudar F, Patel J (2011). Formulation and evaluation of mucoadhesive glipizide films. Acta Pharmaceutica..

[11] Gilhotra RM, Nagpal K, Mishra DN (2011). Azithromycin novel drug delivery system for ocular application. Int. J. pharm. inves..

[12] Aburahma MH, Mahmoud AA (2011). Biodegradable ocular inserts for sustained delivery of brimonidine tartarate: Preparation and in vitro/in vivo evaluation. AAps Pharm. Sci. Tech..

[13] Jain D, Carvalho E, Banerjee R (2010). Biodegradable hybrid polymeric membranes for ocular drug delivery. Acta biomater..

[14] Gilhotra RM, Gilhotra N, Mishra D (2009). Piroxicam bioadhesive ocular inserts: Physicochemical characterization and evaluation in prostaglandin-induced inflammation. Current Eye Rese..

[15] Wong CF, Yuen KH, Peh KK (1999). An in-vitro method for buccal adhesion studies: Importance of instrument variables. Int. J. Pharm..

[16] Jones DS, Pearce KJ (1995). An investigation of the effects of some process variables on the microencapsulation of propranolol hydrochloride by the solvent evaporation method. Int. J. pharm..

[17] Malviya R, Srivastava P, Kulkarni G (2011). Applications of mucilages in drug delivery-a review. Advan. Biological. Res..

[18] Jain RA (2000). The manufacturing techniques of various drug loaded biodegradable poly (lactide-< i> co</i>-glycolide)(plga) devices. Biomater..

[19] Calvo P, Vila-Jato JL, Alonso MaJ (1997). Evaluation of cationic polymer-coated nanocapsules as ocular drug carriers. Int. J. pharm..

[20] Yehia SA, El-Gazayerly ON, Basalious EB (2009). Fluconazole mucoadhesive buccal films: In vitro/in vivo performance. Cur. drug deliver..

[21] Steward P, Hearn J, Wilkinson M (2000). An overview of polymer latex film formation and properties. Advan. colloid and interface sci..

[22] Kusum Devi V, Saisivam S, Maria G, Deepti P (2003). Design and evaluation of matrix diffusion controlled transdermal patches of verapamil hydrochloride. Drug develop. indus. pharm..

[23] Nappinnai M, Chandanbala R, Balaijirajan R (2008). Formulation and evaluation of nitrendipine buccal films. Indian J pharm. sci..

[24] Peptu CA, Ochiuz L, Alupei L, Peptu C, Popa M (2014). Carbohydrate based nanoparticles for drug delivery across biological barriers. J. Biomed. Nanotech..

[25] Sieg JW, Robinson JR (1976). Mechanistic studies on transcorneal permeation of pilocarpine. J. pharm. sci..

[26] Kanikkannan N, Burton S, Patel R, Jackson T, Sudhan Shaik M, Singh M (2001). Percutaneous permeation and skin irritation of jp-8+ 100 jet fuel in a porcine model. Toxicol. let..

